# Association between oral contraceptives with lipid profile: results from Hoveyzeh cohort study (HCS)

**DOI:** 10.1186/s12905-023-02703-7

**Published:** 2023-10-24

**Authors:** Seyed Jalal Hashemi, Rozhan Khezri, Nader Saki, Nahal Nasehi, Seyed Ahmad Hosseini, Mahmood Harizi, Zahra Rahimi

**Affiliations:** 1 Alimentary Tract Research Center, Department of Internal Medicine, School of Medicine, Ahvaz Jundishapur University of Medical Sciences, Ahvaz, Iran; 2 Department of Epidemiology, School of Public Health, Iran University of Medical Sciences, Tehran, Iran; 3 Hearing Research Center, Clinical Sciences Research Institute, Department of Otolaryngology, Head and Neck Surgery, Ahvaz Jundishapur University of Medical Sciences, Ahvaz, Iran; 4 Fertility, Infertility, and Perinatology Research Center, Department of Obstetrics and Gynecology, School of Medicine, Ahvaz Jundishapur University of Medical Sciences, Ahvaz, Iran; 5 Nutrition and Metabolic Diseases Research Center, Clinical Research Institute, Ahvaz Jundishapur University of Medical Sciences, Ahvaz, Iran; 6 Chamran Hospital, Ahvaz Jundishapur University of Medical Sciences, Ahvaz, Iran; 7 Department of Biostatistics and Epidemiology, School of Public Health, Ahvaz Jundishapur University of Medical Sciences, Ahvaz, Iran

**Keywords:** Hyperlipidemia, Oral contraceptives, Dyslipidemia, Iran

## Abstract

**Background:**

Oral contraceptives (OCs) affect lipid metabolism, which can cause hyperlipidemia, a risk factor for cardiovascular diseases. The study was designed to evaluate the possible changes in lipid profile due to using OCs.

**Methods:**

A cross-sectional study was conducted from April 2016 to August 2018 among women from the baseline phase Hoveyzeh cohort study (HCS). Sociodemographic data, anthropometric measurements, physical activity, and biochemical blood tests were measured for every participant. Multiple logistic regression was used to adjust the potential confounders.

**Results:**

Among 2272 participants, 1549 women were OC users, and 723 women were non-user OCs. The mean lipid profile levels were higher in OC users than in non-user OCs. Odds of abnormal Total cholesterol (TC) in OC users were significantly higher than those of non-users OCs [OR = 1.29 (95% CI;1.05 to 1.58)]. Also, the Odds of abnormal low-density lipoprotein (LDL) in OC users was 12% higher than in non-user OCs. However, no significant relationship between abnormal LDL with Oral Contraceptive Pills (OCPs) was observed.

**Conclusions:**

The mean lipid profile was higher in OC users compared to non-user OCs. This finding highlights the need for public health strategies to prevent and detect hyperlipidemia in user OCs.

## Background

Using contraceptives reduces the risk of becoming pregnant; evidence shows that 99% of sexually active women use at least one method of contraception [1]. The OCPs are the most common method of contraception for young women worldwide [2]. One of the reasons for the high adoption of OCPs worldwide is their high performance, accessibility, and ease of use [3].

In Iran, the use of OCPs is the priority of choosing a contraceptive method, and their use rate is about 56% [4, 5]. Due to the widespread use of OCPs, paying attention to their favorable and unfavorable impacts on consumers is necessary. OCPs are divided into combined estrogen-progesterone (COC) or progesterone-only (POP). The most common type is combined hormonal pills (COC) [6]. OCPs interfere with estrogen and progesterone during ovulation and prevent pregnancy by disrupting the normal function of these two hormones. OCPs are primarily used to prevent pregnancy, but in 14% of cases, they are used for other purposes, such as to resolve disorders related to menstruation, menstrual pain, irregular menstruation, fibroids, endometriosis pain, and migraines related to menstruation [1, 7]. Also, some studies showed that OCP consumption reduced even the risk of ovarian, endometrium, and colorectal cancer [8, 9]. Despite the benefits of using OCPs, some evidence is that they can increase the risk of adverse outcomes, including cardiovascular disease(CVD), obesity, high blood pressure, hemorrhagic stroke, breast cancer, and cerebral vein thrombosis [10–13]. Also, some studies showed that the consumption of OCPs can increase oxidative stress. OCPs might be affected through genomic pathways, estrogen receptors, and hepatic apolipoprotein settings or the production of lipids and lipoproteins such as total cholesterol (TC), triglyceride (TG), high-density lipoprotein (HDL), and low-density lipoproteins (LDL) [14].

So far, in some studies, the effect of OCP consumption on lipid profile has been evaluated [15, 16] but their results are contradictory. For example, in the Tehran Lipid and Glucose Study conducted in Iran, no significant differences were observed in cholesterol, triglycerides, and LDL between COC users and non-users [17] while, a study in India showed statistically significant differences in cholesterol, triglycerides, and LDL between the two groups [18]. Despite the importance of dyslipidemia as one of the important risk factors of CVDs, and the common use of contraceptives, only e few studies were conducted to evaluate this issue. Therefore, the present study was investigated in a population-based study to determine the relationship between OCP consumption and lipid profile.

## Methods

### Study design and participants

This study was a population-based cross-sectional study. The participants were women aged 35–70 who entered the Hoveyzeh cohort study (HCS) enrolment phase. The HCS is a population-based cohort study conducted in the Hoveyzeh district that evaluated non-communicable diseases(NCDs) in southwest Iran in 10,009 adults (age 35–70 years) from May 2016 [19]. The Hoveyzeh cohort study center is one branch of the Prospective Epidemiological Research Studies in Iran (the PERSIAN Cohort Study), the biggest population-based cohort study conducted in Iran [20]. Of 5983 women who participated in HCS, 2380 women were in menopause, and 119 women used other hormonal contraceptives that were not eligible for this study. Also, we excluded some possible confounding factors that could confound the relationship between lipid profile and use of oral contraceptive methods, including the subjects who were smokers (n = 65), alcoholics (n = 3), had a history of CVD (n = 366), diabetes (n = 414), renal failures (n = 19) and age ≥ 50 years(n = 345) from the study. Finally, a total of 2272 participants were entered for this analysis. To implement this study, protocols, and questionnaires were taught to the personnel in 8 sessions. The people who got an A grade in the final exam of this course were selected for interview. in this study, the implementation of Quality Assurance (QA) and quality control (QC) teams has been conducted at the central (PERSIAN Cohort) level, the university level, and the local level to guarantee the acquisition of high-quality data. Throughout all phases, ranging from the census to the invitation and enrolment processes, as well as biological sampling, interviewing, examinations, and data entry, diligent observation and supervision are continuously supervised by the quality control teams.

### Definition of covariates

For anthropometric measurements, the participants were wearing light clothes and no shoes. Height was measured to the nearest 0.5 cm by a stadiometer (Seca 206), and weight was measured to the nearest 1 kg using a standing scale (Seca 755). Body mass index (BMI) was calculated as weight (kg) divided by squared height (m2) (< 18.5 = Underweight, 18.5–24.9 = Normal, 25.0-29.9 = Overweight, and ≥ 30 = Obese). We applied the International Physical Activity Questionnaire (IPAQ) to assess the levels of physical activity among the participants based on the Metabolic equivalent of task (MET). A daily physical activity questionnaire was used to measure METs of activity for all participants over a 24-hour day, and quartiles for all participants were calculated [21]. Each participant’s MET score was classified into four quartiles (Q1toQ4) based on the quartile’s classification. The wealth index was computed through the utilization of principle component analysis (PCA) on a total of 14 household assets, such as possession of a refrigerator, personal computer, telephone, mobile phone, washing machine, microwave oven, car, motorcycle, kitchen, bathroom, toilet, house ownership, number of rooms per capita (less than one versus one or more), and the area of the house (below the median versus above the median). Within the realm of principle component analysis, the initial component accounts for the largest proportion of the overall variance. Consequently, assets that were distributed more unequally across the sample population held a greater significance in the first component. The coefficients (weights) assigned to each asset within this initial component were utilized to produce the wealth scores, wherein a higher score corresponded to a higher level of wealth, and vice versa. Lastly, the scores were converted into five consecutive categories, ranging from the poorest to the richest, based on the quintiles, thereby determining the wealth status of each household [22, 23]. Other covariates in this study were education levels (Illiterate, Primary school, Secondary school, High school Diploma, and University), residence type (Urban and Rural), and age groups (35–39, 40–44, and 45–49).

### Oral contraceptive pills

Have you ever used contraceptives including oral contraceptive pills (OCP), cyclofem, LD, HD, medroxyprogesterone acetate, etc.

### Assessment of profile lipids and quality control of the laboratory

In this study, four lipid profiles including LDL, HDL, TC, and TG were evaluated. We took 25 cc of blood from all the participants while fasting from 10 to 12 h before that. Blood samples were centrifuged (Sigma, Germany) at 3000 rpm for 10 min to separate the serum. Then, the lipid profile was measured by a BT 1500 autoanalyzer (Biotecnica Instruments, Italy) [20]. For the laboratory’s quality control, serum samples for normal and pathogen control were established and tested using the BT 1500 device. Subsequently, the results of the control serum were assessed using the Westgard and Levy Jennings quality control chart. The Levy Jennings chart was created with warning limits of x + 2 SD and control limits of x + 3 SD.

### Statistical analysis

The mean and standard deviation (SD) for continuous variables and the number and percent for categorical variables were used to describe the variables. The chi-square test assessed the relationship between categorical variables. The hypothesis of normality for all continuous variables was tested using both the Kolmogorov-Smirnov test and the normal Q-Q plot. We used the independent t-test to compare continuous variables between the two groups. Multiple logistic regression was used to assess the association between oral contraceptives and lipid profile, controlling the confounder factors. Variables with P < 0.25 in the univariate regression analysis were selected to enter the model in multiple analyses. To perform univariate and multiple logistic analysis, the lipid profile as the outcome variables, including LDL ( > = 160 Abnormal, < 160 Normal), TG ( > = 150 Abnormal, < 150 Normal), HDL (< 50 Abnormal, >= 50 Normal), and TC ( > = 200 Abnormal, < 200 Normal) were divided into two categories. The analyses were performed with Stata version 14. The significance level for all the statistical tests was considered less than 0.05.

## Results

Among 2272 participants, 1549(68.2%) of women used OC, whereas 723(31.8%) had no history of OC use. The mean ± SD of the participants’ age were 41.28 ± 2.86. Most OC users were in the age group of 40 to 44 years. The percentage of using OCs decreased with increasing education from illiterate to university. Women who lived in urban areas were more likely to use OCs. The richest participants were more likely to use birth control pills than the poorest individuals. Also, the use of contraceptive pills increased with increasing body mass index and physical activity (Table 1).

**Table 1:** Baseline characteristics of study participants based on using OCs

Variable		Users OCs n (%)	Non-users OCs n (%)	P-value
**Age group**	**35–39**	594(66.5%)	299(33.5%)	0.375
	**40–44**	576(69.6%)	252(30.4%)	
	**45–49**	379(68.8%)	172(31.2%)	
**Education level**	**Illiteracy**	1014(69.6%)	442(30.4%)	0.001^*^
	**Primary school**	325(70.0%)	139(30.0%)	
	**Secondary school**	86(66.2%)	44(33.8%)	
	**High school Diploma**	66(58.9%)	46(41.1%)	
	**University**	58(52.7%)	52(47.3%)	
**Residence Type**	**Urban**	945(70.6%)	393(29.4%)	0.003^*^
	**Rural**	604(64.7%)	330(35.3%)	
**Wealth status**	**Poorest**	282(62.8%)	167(37.2%)	0.002^*^
	**Poor**	333(65.2%)	178(34.8%)	
	**Moderate**	321(68.4%)	148(31.6%)	
	**Rich**	331(73.9%)	117(26.1%)	
	**Richest**	282(71.4%)	113(28.6%)	
**Body Mass Index**	**Underweight**	13(61.9%)	8(38.1%)	0.007^*^
	**Normal**	261(61.7%)	162(38.3%)	
	**Overweight**	510(68.1%)	239(31.9%)	
	**Obese**	765(70.9%)	314(29.1%)	
**Physical activity (MET)**	**Q1**	117(56.3%)	91(43.8%)	< 0.001^*^
	**Q2**	463(66.9%)	229(33.1%)	
	**Q3**	584(69.9%)	252(30.1%)	
	**Q4**	385(71.8%)	151(28.2%)	

*P < 0.05 was considered as a statistically significant level for the chi-square test

BMI: Body Mass Index; MET: Metabolic Equivalent of Task

Table 2 showed that although the means of all lipid profiles, including TG, TC, HDL, and LDL were higher in the user OC group, only the means of cholesterol and HDL were significantly different between the two groups (P > 0.05).

**Table 2:** Comparison of mean lipid profile by use of OCs

Variable	Users OCs	Non-users OCs	t	df	P-value
**TG (mg/dL)**					
Mean	133.58	131.61	-0.597	2270	0.550
SD	71.68	76.59			
**TC (mg/dL)**					
Mean	184.03	180.06	-2.497	2270	0.013^*^
SD	34.98	35.93			
**HDL(mg/dL)**					
Mean	53.51	52.20	-2.388	2270	0.017^*^
SD	12.28	11.82			
**LDL(mg/dL)**					
Mean	103.05	101.58	-1.804	2270	0.710
SD	29.27	29.20			

*P < 0.05 was considered as a statistically significant level for the t-test

TG: Triglyceride; TC: Total Cholesterol; HDL: High-Density Lipoproteins; LDL: Low-Density Lipoprotein

The crude and adjusted odds ratios using the logistic regression model are presented in Tables 3 and 4. The crude odds ratios for all the assessed variables were statistically significant (*p* < 0.25); therefore, we included them in the multiple logistic regression model.

**Table 3:** The crude odds ratios of the assessed factors for lipid profile and their 95% confidence interval using the logistic regression model

Variables		TG	HDL	TC	LDL
		OR Crude (95% CI)	OR Crude (95% CI)	OR Crude (95% CI)	OR Crude (95% CI)
**Age(year)**	35–39	Ref	Ref	Ref	Ref
	40–44	1.16 (0.94–1.43)	1.00 (0.83–1.21)	1.04 (0.84–1.29)	1.02 (0.79–1.32)
	45–49	1.47 (1.15–1.82)^*^	0.80 (0.65–0.99) ^*^	1.44 (1.14–1.82) ^*^	1.27 (0.96–1.68)
**Education levels**	Illiterate	Ref	Ref	Ref	Ref
	Primary school	0.83 (0.69–1.10)	1.33 (1.08–1.64) ^*^	0.76 (0.60–0.96) ^*^	0.92 (0.62–1.22)
	Secondary school	0.65 (0.43–0.99) ^*^	1.41 (0.98–2.02)	0.70 (0.46–1.07)	0.83 (0.50–1.37)
	High school and Diploma	0.83 (0.54–1.27)	2.40 (1.62–3.57) ^*^	0.54 (0.33–0.88) ^*^	0.80 (0.47–1.39)
	University	0.78 (0.50–1.21)	1.04 (0.70–1.54)	0.72 (0.46–1.14)	0.94 (0.56–1.59)
**Residence type**	Urban	Ref	Ref	Ref	Ref
	Rural	0.87 (0.72–1.04)	0.70 (0.59–0.83) ^*^	1.01 (0.84–1.21)	0.91 (0.73–1.14)
**Wealth index**	Poorest	Ref	Ref	Ref	Ref
	Poor	1.12 (0.84–1.48)	1.07 (0.82–1.38)	0.99 (0.75–1.31)	0.97 (0.70–1.37)
	Moderate	1.21 (0.91–1.62)	1.29 (0.99–1.68)	0.84 (0.63–1.13)	1.02 (0.73–1.44)
	Rich	1.39 (1.05–1.86) ^*^	1.41 (1.08–1.83) ^*^	0.91 (0.68–1.22)	0.88 (0.62–1.25)
	Richest	1.18 (0.88–1.60)	1.27 (0.97–1.67)	1.01 (0.75–1.36)	0.91 (0.64–1.32)
**BMI**	Underweight	Ref	Ref	Ref	Ref
	Normal	3.76 (0.50-28.52)	1.84 (0.61–5.59)	2.53 (0.58–11.07)	2.50 (0.33–19.06)
	Overweight	7.54 (1.01–56.51) ^*^	3.02 (1.01–9.06) ^*^	3.90 (0.90-16.89)	4.36 (0.58–32.75)
	Obese	12.21 (1.63–91.31) ^*^	4.18 (1.40–12.50) ^*^	3.99 (0.92–17.22)	4.42 (0.59–33.11)
**Physical activity** **(MET)**	Q1	Ref	Ref	Ref	Ref
	Q2	0.66 (0.48–0.91) ^*^	0.81 (0.59–1.10)	0.97 (0.69–1.36)	1.78 (0.68–4.65)
	Q3	0.60 (0.44–0.82) ^*^	0.73 (0.54–0.99) ^*^	0.94 (0.67–1.32)	1.57 (0.60–4.08)
	Q4	0.64 (0.46–0.90) ^*^	0.65 (0.47–0.90) ^*^	0.89 (0.62–1.27)	1.09 (0.39–3.06)
**Use contraceptive**	Yes	1.09 (0.90–1.33)	0.87 (0.73–1.04)	1.34 (1.09–1.64) ^*^	1.18 (0.93–1.50)
	No	Ref	Ref	Ref	Ref

*P < 0.05 was considered as a statistically significant level in logistic regression

OR: Odds Ratio; CI: Confidence Interval; BMI: Body Mass Index; MET: Metabolic Equivalent of Task; TC: Total cholesterol; TG: Triglyceride; HDL: high-density lipoproteins; LDL: low-density lipoprotein

**Table 4:** The adjusted odds ratios of the assessed factors for lipid profile and their 95% confidence interval using the multiple logistic regression model

Variables		HDL	TC	LDL
		Adjusted OR(95% CI)	Adjusted OR(95% CI)	Adjusted OR(95% CI)
**Age(year)**	35–39	Ref	Ref	Ref
	40–44	1.19 (0.95–1.49)	0.73 (0.58–0.93) ^*^	0.57 (0.32–0.99) ^*^
	45–49	1.21 (0.97–1.52)	0.74 (0.58–0.94) ^*^	0.65 (0.38–1.13)
**Education levels**	Illiterate	Ref	Ref	
	Primary school	1.07 (0.70–1.64)	1.21 (0.76–1.92)	
	Secondary school	1.28 (0.82–1.98)	0.92 (0.56–1.51)	
	High school and Diploma	1.29 (0.76–2.17)	0.86 (0.47–1.58)	
	University	2.35 (1.36–4.07) ^*^	0.70 (0.36–1.34)	
**Residence type**	Urban	Ref		
	Rural	0.83 (0.68–1.01)		
**Wealth index**	Poorest	Ref		
	Poor	1.02 (0.76–1.38)		
	Moderate	1.06 (0.80–1.41)		
	Rich	1.07 (0.81–1.41)		
	Richest	1.23 (0.93–1.63)		
**BMI**	Underweight	Ref	Ref	Ref
	Normal	0.25 (0.08–0.75) ^*^	0.24 (0.06–1.04)	
	Overweight	0.46 (0.36–0.59) ^*^	0.63 (0.48–0.83) ^*^	0.84 (0.43–1.64)
	Obese	0.73 (0.60–0.89) ^*^	0.99 (0.81–1.22)	1.28 (0.78–2.09)
**Physical activity (MET)**	Q1	Ref		Ref
	Q2	1.40 (1.01–1.95) ^*^		0.91 (0.32–2.56)
	Q3	1.14 (0.90–1.44)		1.66 (0.87–3.18)
	Q4	1.07 (0.86–1.34)		1.47(0.77–2.80)
**Use contraceptive**	Yes	0.83 (0. 69-1.01)	1.29 (1.05–1.58) ^*^	1.12 (0.68–1.84)
	No	Ref	Ref	Ref

*P < 0.05 was considered as a statistically significant level in logistic regression

OR: Odds Ratio; CI: Confidence Interval; BMI: Body Mass Index; MET: Metabolic Equivalent of Task; TC: Total cholesterol; HDL: High-Density Lipoproteins

After adjusting for BMI, age, education levels, residence type, wealth index, and physical activity as potential confounders, the odds of abnormal HDL for the BMI group of normal was 4 times less than underweight (reference group) [OR = 0.25 (95% CI; 0.08 to 0.75)]. Also, the odds of having abnormal HDL for university education level was 35% higher than the Illiterate level [OR = 2.35 (95% CI; 1.36 to 4.07)]. Also, the odds ratio of abnormal HDL was 40% higher among women with moderate physical activity than participants with low physical activity [OR = 1.40 (95% CI; 1.01 to 1.95)].

After adjusting for BMI, age, and education levels as potential confounders, the women users of oral contraceptives had 29% more odds of abnormal TC than those non-users of oral contraceptives [OR = 1.29 (95% CI;1.05 to 1.58)]. Also, the odds of abnormal TC for the BMI group Overweight was 37% less than that of the underweight group as the reference group [OR = 0.63 (95% CI;0.46 to 0.59)]. The odds of abnormal TC for the age group 40–44 and 45–49 were 27% [OR = 0.73(95% CI;0.58 to 0.93)] and 26% [OR = 0.74(95% CI;0.58 to 0.94)] less than the age group 30 to 35.

After adjusting for BMI, age, and physical activity as potential confounders, the odds of abnormal LDL for the age group 40–44 was 43% less than that of the age group 30–35 as the reference group [OR = 0.57(95% CI;0.32 to 0.99)].

## Discussion

This study aims to evaluate the association between the use of oral contraceptives with lipid profile. The finding showed a significant relationship between OC use and abnormal TC. The OC users had significantly higher odds of abnormal TC than those non-users. Despite the higher mean lipid profile in OC users, the relationship between OC use with abnormal HDL, abnormal LDL, and abnormal triglyceride was not statistically significant.

Because of the widespread use of contraceptive methods, even a slight increase in the odds of an abnormal lipid profile or increase in mean TG in OCs users compared to non-users could be clinically meaningful. Our findings showed a considerably high use of OCs in the studied population (68.18%).

The present study found higher TG levels in the serum of OC users. A similar rise in TG levels was observed previously [16, 24, 25]. Estrogen increases lipogenesis, resulting in increased TG levels. Elevated triglyceride levels are considered a marker of cardiometabolic disease, and it has been suggested that long-term use of OCs may increase the risk of acute metabolic syndrome. Despite this important clinical finding, however, the findings of different studies are inconsistent. Some similar studies found no association between abnormal TG and OC use [26, 27]. However, several studies reported a statistically significant association between abnormal TG and the use of OCs [18, 28].

Our results showed the odds of abnormal HDL were not statistically significant in user OCs compared to non-user OCs. The progesterone component of hormonal contraceptives increases the activity of the hepatic lipase enzyme, which increases the removal of HDL, thereby reducing the serum HDL level [12, 29]. The association of users of OCs with abnormal HDL is inconsistent in the results of various studies. Some studies similar to our results found no relationship between OC use with abnormal HDL [18, 30]. On the other hand, some studies have shown OC use was significantly associated with increased HDL [12, 31]. Our results demonstrated that after controlling the confounders in multivariable regression analysis, BMI, education levels, and physical activity were significantly associated with abnormal HDL. So the odds of abnormal HDL in normal BMI, overweight, and obese groups were 75%, 54%, and 27% less than underweight groups, respectively. The association of abnormal HDL with BMI is indicated in various studies [12, 31, 32]. According to multivariable analysis, the odds of having abnormal HDL were two times higher in women with a university education level compared with those who were illiterate. Previous studies reported an inverse relationship between abnormal HDL-C and SES [33]. A recent study has shown the correlation between physical activity and lipid profile indices [34]. Our findings showed that the odds of having abnormal HDL in women with moderate physical activity were 40% higher than in those with low physical activity.

The univariate results showed the odds of abnormal TC in OCs users were 34% higher than in non-users of OCs. After controlling the confounding variables in a multivariable regression analysis, the association between OC use and abnormal TC remained statistically significant. So, the odds of abnormal TC are 29% higher in OCs users than in non-user OCs. This result was in line with the majority of research conducted in Iran and other countries [18, 28, 35]. The abnormal increase in TC is a known risk factor for atherosclerosis and coronary artery disease [36].

This study showed that the odds of abnormal LDL in users of OCs were higher than in non-users of OC, but this association was not significant. The previous studies indicated the odds ratio of hypercholesterolemia was significantly higher in women who used OCPs than in non-users of OCP [18, 28, 37]. The abnormal increase in LDL can cause atherosclerosis and coronary artery disease [38]. The multiple logistic regression analysis findings showed that the variables significantly associated with abnormal LDL included age. However, the relationship between physical activity and abnormal LDL was not statistically significant. Conversely, in a similar study, the relationship between physical activity and LDL was statistically significant. They reported that aerobic exercise has a beneficial effect on the composition of LDL [39].

### Strengths and limitations of the study

Our study’s Strengths included; First, our research encompassed a large sample size and was carried out within the framework of a cohort study that focused on the general population. This approach allowed us to obtain more accurate findings by minimizing the likelihood of random error. Second, the use of well-trained interviewers and multiple levels of supervisors. Third, the outcome data were measured using standard and calibrated devices and by laboratory experts. However, the cross-sectional design was the limitation of our study; the associations that were observed do not establish causality due to the study design being a cross-sectional survey, which allows for the potential bias of reverse causality.

## Conclusion

The results of our study showed that the average lipid profile was higher in user OCs, while the relationship between OCs use and lipid profile was statistically significant only in TC. This finding is clinically important, and it is recommended to periodically check the lipid profile in user OCs so that in the event of a disturbance in the lipid profile, another contraceptive method can be replaced as soon as possible to prevent cardiovascular diseases in these women. Also, user OCs should be advised to eat a balanced diet and pay attention to proper physical activity. On the other hand, to enhance women’s health, men can be encouraged to participate in contraception, which can partially prevent the adverse effects of contraceptive methods in women, including increased lipid profile. A comprehensive longitudinal study of sufficient duration, considering the duration of OC consumption for various OCP types, is needed.

## Data Availability

The datasets provided during and analyzed during this study are available from the corresponding author upon reasonable request.

## Abbreviations

OCs: Oral Contraceptives
OCPs: Oral Contraceptive Pills
LD: Low-Dose Contraceptives
HD: High-Dose Contraceptives
COC: Combined Oral Contraceptives or Combined hormonal pills
POP: Progesterone-Only Pill
TC: Total cholesterol
TG: Triglyceride
HDL: High Density Lipoproteins
LDL: Low-Density Lipoprotein
BMI: Body Mass Index
CVDs: Cardiovascular Diseases
NCDs: Non-Communicable Diseases
QA: Quality Assurance
QC: Quality Control
PCA: Principle Component Analysis
IPAQ: International Physical Activity Questionnaire
MET: Metabolic Equivalent of Task
